# Effects of correlated collisions and intermittency on the growth of lucky droplets

**DOI:** 10.1073/pnas.2502553123

**Published:** 2026-02-23

**Authors:** Tobias Bätge, Johannes Zierenberg, Michael Wilczek

**Affiliations:** ^a^Max Planck Institute for Dynamics and Self-Organization, Göttingen 37077, Germany; ^b^Faculty of Physics, University of Göttingen, Göttingen 37077, Germany; ^c^Theoretical Physics I, University of Bayreuth, Bayreuth 95440, Germany

**Keywords:** turbulence, particle-laden flows, cloud microphysics, intermittency, non-Markovian

## Abstract

To trigger precipitation, water droplets in warm clouds need to attain a sufficient size. Theoretical estimates based on condensation and gravitational collisions alone fail to explain the observed timescales for the onset of precipitation for a range of droplet sizes. This suggests the involvement of collisional growth mediated by turbulence to resolve the so-called “size-gap problem.” For the onset of rain, it is sufficient that statistical outliers, coined “lucky droplets,” cross the size gap. In this study, we explore the influence of turbulence on droplet growth, focusing on correlated collisions and intermittency. Using direct numerical simulations of droplets in turbulent flow, we constrain a non-Markovian stochastic framework that allows us to assess memory effects on the droplet-size distribution arising from correlations between consecutive collisions. Using our framework, we find that correlated collisions accelerate the initial growth of lucky droplets but have subleading effect at later stages. Consequently, we neglect correlations from collisions and model an ensemble of cloud parcels representing fluctuations in the volume-averaged dissipation rate. Here, the distribution of droplet sizes in each parcel is described by a linear master equation with a time-dependent collision rate according to the volume-averaged dissipation rate. Our analyses of this toy model show that intermittency can significantly reduce the time required by lucky droplets to cross the size gap.

## Significance

Given the complexity of precipitation in warm clouds, simple conceptual models are crucial for identifying key aspects of accelerated droplet growth. Statistical outliers with anomalously frequent collisions, so-called lucky droplets, are commonly assumed to play a central role in explaining the timescales of the onset of precipitation. Here, we investigate how the formation of lucky droplets is accelerated by correlated collisions and turbulence intermittency. Using a non-Markovian stochastic framework, we show that while short-term correlations enhance early growth, dissipation fluctuations dominate the process. Our approach, which models a statistical ensemble of cloud parcels based on turbulent dissipation rates, provides a systematic path for bridging scales between simulations or laboratory studies and atmospheric clouds.

In warm clouds, the so-called “size gap” constitutes a major open problem in the formation of rain droplets (1, 2). While droplets smaller than 15μm spontaneously form due to condensation (3), and droplets larger than 50μm rapidly grow due to gravity-induced differential settling (4), it remains a subject of investigation how droplets can grow from 15 to 50μm and bridge the size gap within the typical timescale for rain onset of about 30 min (2).

Here, turbulence is believed to be vital by amplifying growth through collisions (5). It may introduce spatial clustering (6–8) and high relative velocities (9, 10), where also the so-called sling effect (11, 12) contributes to the collision kernel (11, 13, 14). However, even with turbulence the average time between collisions of small droplets is typically on the order of hours and thus too long to explain rain onset (4).

An elegant explanation of rapid rain formation despite slow average collision events is through statistical fluctuations (15, 16). As collisions are a random process, there is a small probability to observe the rare event of fast-growing droplets that were initially called “fortunate” (15) and later coined “lucky droplets” (17). Previous results on these rare events (17–19) imply that lucky growth in turbulence could be a crucial ingredient to explain rain initiation.

However, these estimates assume collision rates to be constant with time, while collisions occur preferably in distinct regions of the flow (20) and successive inertial particle collisions are known to be correlated (6, 21). By means of a generalized collision–coalescence framework, such correlated collisions have been shown to accelerate particle growth (21). Additionally, spatiotemporal fluctuations of the volume-averaged dissipation rate render collision rates time dependent as droplets pass through regions of varying dissipation rates (22). Both aspects could further accelerate the growth of lucky droplets, and we here aim to investigate their effect under representative turbulence conditions in warm clouds.

Cloud conditions involve high-Reynolds-number turbulence that comes with a large separation between the largest and smallest spatiotemporal scales in the flow along with strong intermittency. Intermittency manifests itself in extreme spatiotemporal fluctuations of the dissipation rate. The statistics of these fluctuations can be captured by various modeling approaches such as the refined similarity hypothesis (K62) (23, 24), the β-model (25), as well as multifractal models (26–28), a corresponding large-deviation formulation (29), or a model which accounts for observed deviations of log-normality (30). These fluctuations are often accounted for only on the scales attainable by simulations. However, with the extreme range of scales present in clouds, those fluctuations are more pronounced and might also lead to even more pronounced fluctuations in droplet growth and thus more extreme statistical outliers, i.e., lucky droplets.

In this study, we investigate the growth of lucky droplets in a turbulent flow and incorporate spatiotemporal fluctuations in the volume-averaged dissipation rate through an ensemble model. Using direct numerical simulations of cloud parcels with fixed volume-averaged dissipation, we find that strong dissipation can induce correlated collisions that may accelerate droplet growth on short timescales but turn out to be a subleading correction at later times. This is consistent with previous reasoning (17–19). Based on our numerical results, we parameterize a simple toy model of collisional growth in a cloud parcel with a time-dependent volume-averaged dissipation rate to model temporal fluctuations. Subsequently, we evaluate collisional growth in an ensemble of cloud parcels, modeling spatiotemporal fluctuations, to find that such fluctuations of the volume-averaged dissipation can substantially accelerate the formation of lucky droplets.

## Model and Methods

We model a parcel of cloud turbulence by direct numerical simulations (DNS) of Navier–Stokes turbulence. While turbulence in clouds spans multiple scales, from the integral scale L∼100m down to the Kolmogorov length scale $\eta_{K}∼1\;\;\text{mm}$ (31), our simulations are limited to length scales of $O\left(1\;\;\text{m}\right)$ and times scales of $O\left(1\;\;\text{s}\right)$ where we approximate the flow by homogeneous isotropic turbulence. We consider the DNS box as a representation of a small cloud parcel, see, e.g., refs. 32, 33, 34, 35. The key idea for the following analysis is to take into account dissipation fluctuations on scales larger than the individual simulation box through ensemble modeling. This approach offers a complementary perspective to Large Eddy Simulations, where the large scales are explicitly resolved and the small scales are modeled.

On the length scale of our simulation box, we estimate the amplitude of dissipation fluctuations with the refined similarity hypothesis (23, 24) that was shown to reasonably describe intermittent features in in-situ measurements, experiments, and simulations (36, 37). In this case, the volume-averaged dissipation rate $\epsilon_{r}$ averaged over a sphere of radius r (or box of side length r in our case) is log-normally distributed (23, 24):

[1] $$\begin{array}{c} P\left({\overset{¯}{\epsilon }}_{r}\right)=\frac{1}{{\overset{¯}{\epsilon }}_{r}\sigma \sqrt{2\pi }}\exp\left(-\frac{{\left(\ln{\overset{¯}{\epsilon }}_{r}+\sigma^{2}/2\right)}^{2}}{2\sigma^{2}}\right)\;, \end{array}$$

where ${\overset{¯}{\epsilon }}_{r}=\epsilon_{r}/\left\langle \epsilon \right\rangle$ is the averaged dissipation rate rescaled by the global mean, and the variance is determined by the length-scale ratio L/r,

[2] $$\begin{array}{c} \sigma^{2}=\;\ln A+\mu \ln\frac{L}{r}\;\;\text{with}\;\left\langle {\overset{¯}{\epsilon }}_{r}^{2}\right\rangle =A{\left(\frac{L}{r}\right)}^{\mu }. \end{array}$$

**Fig. 1.**
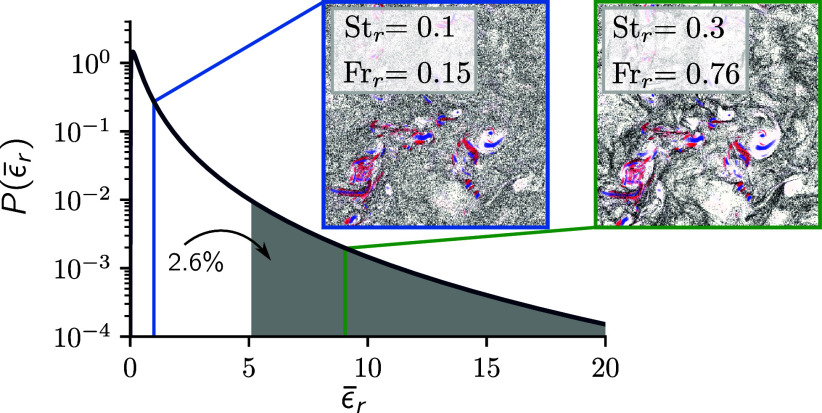
Intermittency in clouds causes strong fluctuations of the normalized dissipation rate ${\bar{\varepsilon }}_{r}$ averaged over a volume of extent r, here modeled with a log-normal distribution (see text for details). As a result, parameters that depend on the average dissipation rate, such as the Stokes number of droplets of a given size and the Froude number, vary locally across a cloud. We can mimic this in numerical simulations by varying these parameters, see *Insets*, which show typical simulation snapshots for $\epsilon_{r}=\left\langle \epsilon \right\rangle$ (blue frame) and $\epsilon_{r}=9\left\langle \epsilon \right\rangle$ (green frame), where particles are shown in black, regions of strong vorticity in blue, and regions of strong strain in red.

Here, μ≈0.25 (38) and A is an order-one constant that depends on the large scales and, in the following, is set to A=1 for simplicity. Note that the log-normal distribution of the dissipation rate—while being a reasonable model approximation—is not a fully accurate description as there are deviations from the log-normal distribution, especially for high Reynolds numbers, see, e.g., ref. 30. The heavy-tailed nature of $P({\overset{¯}{\epsilon }}_{r})$ implies that simulation parcels can have a very high volume-averaged dissipation rate (Fig. 1). For the example of an integral scale L=100m and a cloud parcel on the scale of r=0.25m [this corresponds to our simulation box for a high $\epsilon_{r}$, see *SI Appendix* (39) for more details on the simulation setup], we find that the 2.6% most dissipative cloud parcels have on average a volume-averaged dissipation rate that is nine times larger than the global mean (${\overset{¯}{\epsilon }}_{r}=1$).

Within the turbulent flow of each simulation, we model droplets as spherical Stokes particles. The droplet position x(t) and velocity v(t) change according to Stokes drag and gravity (40)

[3] $$\begin{array}{c} \dot{x}\left(t\right)=v\left(t\right)\;\dot{v}\left(t\right)=\frac{1}{\tau_{d}}\left[u\left(x\left(t\right),t\right)-v\left(t\right)\right]+ge_{z}\;, \end{array}$$

where u denotes the turbulent flow velocity, g the gravitational acceleration and $\tau_{d}=\frac{2}{9}\frac{a^{2}}{\nu }\frac{\rho_{\text{d}}}{\rho_{\text{f}}}$ is the particle response time. Here, a denotes the droplet radius, ν the kinematic viscosity, and $\rho_{\text{d}}$ and $\rho_{\text{f}}$ denote the density of the droplet and surrounding fluid, respectively. This neglects terms from added mass or history forces, which become small due to a large density ratio between water and air as well as the small droplet radius compared to the Kolmogorov length, cf. (4, 40). If two droplets collide, they merge, i.e., we assume a collision efficiency of one; see *SI Appendix* (39) for more details on the collision procedure.

The system of the turbulent flow and Stokes particles is characterized by three parameters: the Stokes number $\text{St}=\tau_{d}/\tau_{K}$ quantifies the relevance of inertial effects. Here, $\tau_{K}=\sqrt{\nu /\langle \epsilon \rangle }$ is the Kolmogorov time determined from the mean dissipation rate and the kinematic viscosity. The Froude number $\text{Fr}=a_{K}/g$ quantifies the relevance of gravity, where $a_{K}=\eta_{K}/\tau_{K}^{2}$ is the Kolmogorov acceleration and $\eta_{K}={\left(\nu^{3}/\left\langle \epsilon \right\rangle \right)}^{1/4}$ the Kolmogorov length. The last parameter is the number density relative to the Kolmogorov length $\rho_{\text{N}}=N/\eta_{K}^{3}$. The Kolmogorov scales are determined by the mean dissipation rate. For our analysis, we also define the local Kolmogorov scales based on the volume-averaged dissipation rate, i.e., $\tau_{K,r}=\sqrt{\nu /\epsilon_{r}}$, $\eta_{K,r}={\left(\nu^{3}/\epsilon_{r}\right)}^{1/4}$, and $a_{K,r}=\eta_{K,r}/\tau_{K,r}^{2}$. Therefore, fluctuations in ${\overset{¯}{\epsilon }}_{r}$ directly affect the local Stokes number ${\text{St}}_{r}=\tau_{d}/\tau_{K,r}$ and Froude number ${\text{Fr}}_{r}=a_{K,r}/g$ on the scale of cloud parcels, see Fig. 1.

As collisions are a random process, collisional growth calls for a statistical description. The following general master equation describes the time-dependent probability to find droplets of size n

[4] $$\begin{array}{cc} {\dot{P}}_{n}\left(t\right) & =j_{n}^{\text{in}}\left(t\right)-j_{n}^{\text{out}}\left(t\right)\;, \end{array}$$

where $j_{n}^{\text{in}}\left(t\right)$ and $j_{n}^{\text{out}}\left(t\right)$ are influxes and outfluxes, respectively, to be specified. In the Markovian case of memoryless collisions, one can describe these fluxes by constant collision kernels between n and $n^{\prime }$ within the established Smoluchowski equation (2, 4). For our analytical computations, we assume that droplets most likely collide with droplets of their initial size, i.e., $n^{\prime }=1$. As a result, droplet size becomes a discrete variable n>0 that increases in integer steps, leading to a direct relation between influx and outflux, $j_{n+1}^{\text{in}}=j_{n}^{\text{out}}$. In addition, we assume that droplets grow effectively in a statistically stationary background distribution, i.e., the reservoir of background droplets is not reduced by collisions. As a result, the Smoluchowski equation is linear in the evolving droplet distribution. This translates to a simple master equation with constant collision rates $\lambda_{n}$ (15)

[5] $$\begin{array}{cc} {\dot{P}}_{n}^{\text{M}}\left(t\right) & =\lambda_{n-1}P_{n-1}^{\text{M}}\left(t\right)-\lambda_{n}P_{n}^{\text{M}}\left(t\right)\;, \end{array}$$

where the superscript M denotes the Markovian case. Using variation of constants one obtains a recursive relation for $P_{n}^{\text{M}}$ that can be expressed for our initial condition $P_{n}\left(0\right)=\delta_{n1}$ as [*SI Appendix* (39)]

[6] $$\begin{array}{cc} P_{n}^{\text{M}}\left(t,\lambda \right) & =\int_{-\infty }^{t}dt_{n}\;\cdots \int_{-\infty }^{t_{2}}dt_{1}\;\delta \left(t_{1}\right)\\ & \times \prod_{i=1}^{n-1}\left[\lambda_{i}e^{-\lambda_{i}\left(t_{i+1}-t_{i}\right)}\right]e^{-\lambda_{n}\left(t-t_{n}\right)}, \end{array}$$

where the tuple $\lambda =(\lambda_{1},...,\lambda_{n})$ specifies the constant collision rates of droplets up to size n. This can also be solved explicitly to yield (15)

[7] $$\begin{array}{cc} P_{n}^{\text{M}}(t,\lambda ) & =\sum_{l=1}^{n-1}\left[\frac{\prod_{i=1}^{n-1}\lambda_{i}}{\prod_{\begin{array}{l} i=1\\ i\neq l \end{array}}^{n}(\lambda_{i}-\lambda_{l})}\left(e^{-\lambda_{l}t}-e^{-\lambda_{n}t}\right)\right], \end{array}$$

where we assume that different collision rates are not identical, i.e., $\lambda_{i}\neq \lambda_{l}$. This Markovian solution assumes that collision times are uncorrelated, an approximation that is not necessarily true in turbulence, as previously shown in ref. 21.

To investigate to what extent this assumption holds in cloud conditions, we turn to DNS. For our numerical investigations, we use the pseudospectral fluid solver TurTLE (41). We consider different dissipation rates that are expected to occur in cloud conditions. To increase the statistics of correlated collisions in our simulations, we choose a physical droplet number density $1,000\;\;{\text{cm}}^{-3}$ at the upper limit of what might occur in clouds [see *SI Appendix* (39) for a discussion of how simulation parameters relate to the physical characteristics of clouds (31)]. In the main text, we focus on the mean volume-averaged dissipation rate ${\overset{¯}{\epsilon }}_{r}=1$ (${\text{St}}_{r}=0.1$, ${\text{Fr}}_{r}=0.15$, $\rho_{N}=0.18\;N\eta_{K,r}^{-3}$), as well as a large volume-averaged dissipation rate ${\overset{¯}{\epsilon }}_{r}=9$ (${\text{St}}_{r}=0.3$, ${\text{Fr}}_{r}=0.76$, $\rho_{N}=0.035\;N\eta_{K,r}^{-3}$) to characterize the strong fluctuations in the 2.6% most-dissipative parcels.

## The Effect of Short-Time Correlations

To quantify the effect of correlations, we measure the conditional collision rate $\lambda_{n}\left(\tau \right)$ for all droplets of size n for which a collision has occurred at τ=0. This conditional collision rate is connected to the survival probability (see, e.g., ref. 42)

**Fig. 2.**
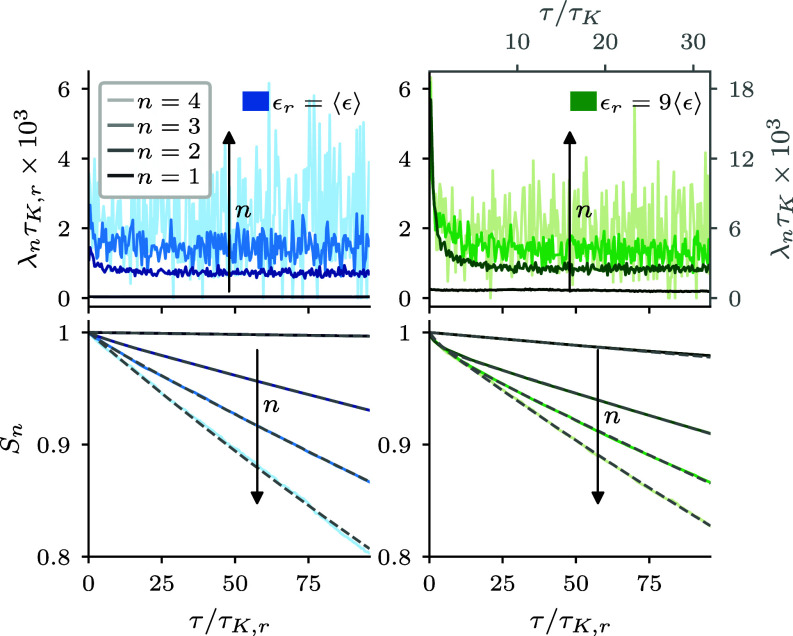
Collisions in turbulence are non-Markovian but can be approximated as a superposition of Poisson processes with different timescales. Data from DNS (see *SI Appendix* for parameters) with normalized volume-averaged dissipation rate ${\overset{¯}{\epsilon }}_{r}=1$ (blue) and ${\overset{¯}{\epsilon }}_{r}=9$ (green). (*Top*) Conditional collision rate for droplets of size n. The increase at short times implies temporal correlations and violates the memoryless assumption of Poisson processes with a constant rate. (*Bottom*) The survival probability can be well approximated by a sum of two exponential functions (gray dashed lines). Note that we nondimensionalized with the local Kolmogorov time $\tau_{K,r}$. On the left, this is identical to mean conditions $\tau_{K}$. On the right, the local and global Kolmogorov times are different and we show the additional axes nondimensionalized by $\tau_{K}$ in gray.

[8] $$\begin{array}{c} S_{n}\left(\tau \right)=e^{-\int_{0}^{\tau }\lambda_{n}\left(\tau^{\prime }\right)d\tau^{\prime }}\;\;\text{for}\;\tau \geq 0\;, \end{array}$$

which quantifies the fraction of droplets of size n that have not collided again by time τ (Fig. 2). If consecutive collisions are not correlated, then $\lambda_{n}\left(\tau \right)=\lambda_{n}$ is constant, which results in exponential survival probabilities $S_{n}\left(\tau \right)=e^{-\lambda_{n}\tau }$. Since we prepare the system initially with particles of size n=1, the first collision cannot be correlated to a previous collision. This implies that $\lambda_{1}$ is—except for small effects of a slowly reducing number of droplets—constant. Our analyses further reveal that for the global mean, ${\overset{¯}{\epsilon }}_{r}=1$, there are correlations in the collision rates with a slight increase of $\lambda_{n}\left(\tau \right)$ at very short times of $O(\tau_{K,r})$. These correlations, though, have no notable effect on $S_{n}\left(\tau \right)$ (Fig. 2, *Left*). By dimensional arguments, one may expect that in terms of the local Kolmogorov units the results are similar for higher volume-averaged dissipation rates. Instead, we find for large dissipation, here ${\overset{¯}{\epsilon }}_{r}=9$, a stronger time dependence in $\lambda_{n}\left(\tau \right)$ and deviations from the exponential shape of $S_{n}\left(\tau \right)$. This points to the increased relevance of inertial effects such as clustering, (Fig. 1) and implies impactful correlations between collision times of $O(10\tau_{K,r})$ (Fig. 2, *Right*). We can capture the correlations by approximating the survival probability by a superposition of two exponentials, i.e., the superposition of two Poisson processes with different (constant) collision rates,

[9] $$\begin{array}{c} S_{n}\left(\tau \right)=A_{n}^{\mathrm{slow}}e^{-\lambda_{n}^{\mathrm{slow}}\tau }+A_{n}^{\mathrm{fast}}e^{-\lambda_{n}^{\mathrm{fast}}\tau }\;, \end{array}$$

where $A_{n}^{\mathrm{slow}}+A_{n}^{\mathrm{fast}}=1$ (see *SI Appendix* for more details on fitting parameters). We thus find evidence of correlations that violate the assumptions underlying Eq. **5**. What remains to be quantified is whether they are relevant to droplet growth.

To investigate the relevance of correlations, we use a non-Markovian stochastic framework similar to the one established in ref. 21 that allows us to include correlations in our statistical description explicitly.^*^ Due to the time-dependent collision rates, we cannot start from the Markovian master equation, Eq. **5**, but need to derive a new master equation and corresponding solution for droplet growth from monodisperse initial conditions with non-Markovian influx and outflux.

Let us begin with the special case of droplets of size n=1. Since we prepare the system with a monodisperse droplet distribution at time $t_{0}=0$, i.e., $P_{n}\left(0\right)=\delta_{n,1}$ and $P_{1}\left(0\right)=1$, the probability of finding droplets of size n=1 is directly given by the survival probability $P_{1}\left(t\right)=S_{1}\left(t-t_{0}\right)$. For n=1, an influx occurs only during preparation as an initial condition, i.e., $j_{1}^{\text{in}}\left(t\right)=\delta \left(t-t_{0}\right)$, such that all later changes of probability ${\dot{P}}_{1}\left(t\right)$ in Eq. **4** are created by the outflux, i.e., $j_{1}^{\text{out}}\left(t\right)=-{\dot{S}}_{1}\left(t-t_{0}\right)$. From Eq. **8** follows that $j_{1}^{\text{out}}\left(t\right)=S_{1}\left(t-t_{0}\right)\lambda_{1}\left(t-t_{0}\right)$, which reveals that the outflux is determined by the (time-dependent) collision rate of those droplets that entered state n=1 at time $t_{0}$ and survived until time t.

We can generalize this intuition to the case n>1. As we noticed for n=1, the outflux is determined by the collision rate of droplets that entered at time $t_{n}$ and survived until time t, which corresponds to $S_{n}\left(t-t_{n}\right)\lambda_{n}\left(t-t_{n}\right)=-{\dot{S}}_{n}\left(t-t_{n}\right)$. This needs to be weighted with all past influxes to yield

[10] $$\begin{array}{c} j_{n}^{\text{out}}\left(t\right)=-\int_{-\infty }^{t}j_{n}^{\text{in}}\left(t_{n}\right){\dot{S}}_{n}\left(t-t_{n}\right)dt_{n}\;. \end{array}$$

Notice that for n=1, we obtain an integral over influx times $t_{1}$ that reduces to our above expression due to $j_{i}^{\text{in}}\left(t_{1}\right)=\delta \left(t_{1}-t_{0}\right)$. For $t_{0}=0$, we can insert the initial condition into Eq. **10**, and by using $j_{n}^{\text{in}}\left(t\right)=j_{n-1}^{\text{out}}\left(t\right)$ we iteratively find

[11] $$\begin{array}{cc} j_{n}^{\text{in}}\left(t_{n}\right)= & \int_{-\infty }^{t_{n}}dt_{n-1}\;\cdots \int_{-\infty }^{t_{2}}dt_{1}\;\delta \left(t_{1}\right)\left[\prod_{i=1}^{n-1}-{\dot{S}}_{i}\left(t_{i+1}-t_{i}\right)\right]. \end{array}$$

Using Eq. **10** in the master equation, Eq. **4**, it generalizes for non-Markovian collision rates to the form

[12] $$\begin{array}{cc} {\dot{P}}_{n}\left(t\right) & =j_{n}^{\text{in}}\left(t\right)+\int_{-\infty }^{t}j_{n}^{\text{in}}\left(t_{n}\right){\dot{S}}_{n}\left(t-t_{n}\right)dt_{n}\;. \end{array}$$

This is solved by

[13] $$\begin{array}{cc} P_{n}\left(t\right) & =\int_{-\infty }^{t}\;j_{n}^{\text{in}}\left(t_{n}\right)S_{n}\left(t-t_{n}\right)dt_{n}\;, \end{array}$$

as one can verify by inserting back into Eq. **12** and applying the Leibniz integral rule to evaluate the time derivative on the left-hand side. Moreover, this solution connects to our intuition that the probability of finding droplets in state n is determined by integrating over the past influx from state n−1 that survived until time t.

Also, note that the evolution equation Eq. **12** reduces to Eq. **5** for constant collision rates. To see that we take an exemplary look at the outflux $j_{n}^{\text{out}}$, see Eq. **10**, where we can perform the derivative ${\dot{S}}_{n}$ to pull out the now constant collision rate

[14] $$\begin{array}{cc} j_{n}^{\text{out}}\left(t\right)= & -\int_{-\infty }^{t}j_{n}^{\text{in}}\left(t_{n}\right){\dot{S}}_{n}\left(t-t_{n}\right)dt_{n}\;, \end{array}$$

[15] $$\begin{array}{cc} = & \lambda_{n}\int_{-\infty }^{t}j_{n}^{\text{in}}\left(t_{n}\right)S_{n}\left(t-t_{n}\right)dt_{n}=\lambda_{n}P_{n}\left(t\right)\;. \end{array}$$

In the last step, we identified Eq. **13**. As $j_{n}^{\text{in}}=j_{n-1}^{\text{out}}$, this formulation agrees with Eq. **5**, showcasing the reduction for constant collision rates.

To solve Eq. **13** with its recursive convolutions, we generalize our empirical observation that the survival probability can be expressed as a superposition of exponentials, Eq. **9**. Taking a continuum of exponentials yields the Laplace transform

[16] $$\begin{array}{c} S_{n}\left(\tau \right)=\int_{0}^{\infty }{\hat{S}}_{n}\left({\hat{\lambda }}_{n}\right)e^{-{\hat{\lambda }}_{n}\tau }d{\hat{\lambda }}_{n}\;. \end{array}$$

where ${\hat{S}}_{n}\left({\hat{\lambda }}_{n}\right)$ corresponds to the weight of a Poisson process with constant collision rate ${\hat{\lambda }}_{n}$. Using the superposition Eq. **16**, one can explicitly solve the convolution integrals (stemming from the iterative pattern of the fluxes Eq. **10**) such that the solution Eq. **13** becomes

[17] $$\begin{array}{cc} P_{n}\left(t\right) & =\int_{0}^{\infty }d\lambda_{n}\;\cdots \int_{0}^{\infty }d\lambda_{1}\prod_{i=1}^{n}{\hat{S}}_{i}\left({\hat{\lambda }}_{i}\right)\\ & \times \int_{-\infty }^{t}dt_{n}\;\cdots \int_{-\infty }^{t_{2}}dt_{1}\;\delta \left(t_{1}\right)\\ & \times \prod_{i=1}^{n-1}\left[{\hat{\lambda }}_{i}e^{-{\hat{\lambda }}_{i}\left(t_{i+1}-t_{i}\right)}\right]e^{-{\hat{\lambda }}_{n}\left(t-t_{n}\right)}, \end{array}$$

[18] $$\begin{array}{cc} & =\int_{0}^{\infty }d\lambda_{n}\;\cdots \int_{0}^{\infty }d\lambda_{1}\prod_{i=1}^{n}{\hat{S}}_{i}\left({\hat{\lambda }}_{i}\right)P_{n}^{M}\left(t,\hat{\lambda }\right)\;, \end{array}$$

where we identified the Markovian solution within the integral, cf. Eq. **6**. Hence, the solution to Eq. **12** with memory effects becomes a superposition of solutions without memory effects. This also provides a procedure to turn correlations on and off by choosing the number of Poisson processes in Eq. **16**. Since collisions in our simulations are well captured by the superposition of two Poisson processes (Fig. 2), we can use Eq. **18** by inserting

[19] $$\begin{array}{c} {\hat{S}}_{n}\left(\hat{\lambda }\right)=A_{n}^{\text{slow}}\delta \left(\hat{\lambda }-\lambda_{n}^{\text{slow}}\right)+A_{n}^{\text{fast}}\delta \left(\hat{\lambda }-\lambda_{n}^{\text{fast}}\right)\;. \end{array}$$

This leads to a solution with memory effects as a sum of the known solutions $P_{n}^{\text{M}}$ for all possible combinations of constant collision rates:

[20] $$\begin{array}{cc} P_{n}\left(t\right)= & \left[\prod_{i=1}^{n}\left(A_{i}^{\text{fast}}\delta_{{\hat{\lambda }}_{i},\lambda_{i}^{\text{fast}}}+A_{i}^{\text{slow}}\delta_{{\hat{\lambda }}_{i},\lambda_{i}^{\text{slow}}}\right)\right]P_{n}^{M}\left(t,\hat{\lambda }\right)\;, \end{array}$$

where we can easily deactivate correlations for any i by setting $A_{i}^{\text{fast}}=0$ and $A_{i}^{\text{slow}}=1$.

**Fig. 3.**
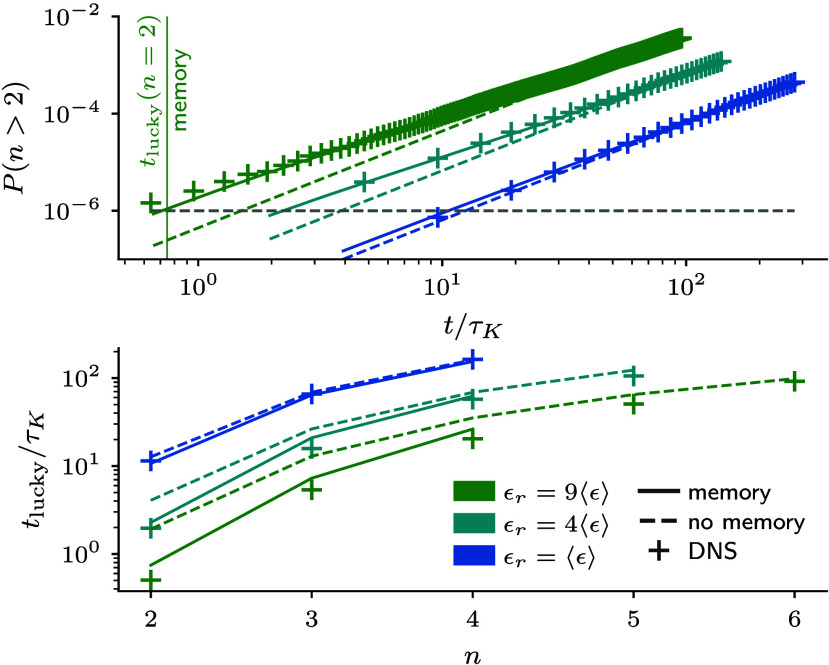
(*Top*) Probability of finding droplets of size n>2: We compare our non-Markovian stochastic framework using the empirical fit to $S_{n}$ to turning off correlations by removing the fast-decreasing exponential and our DNS data of droplet growth. This shows that memory effects can further accelerate growth at short times. In the case of $\epsilon_{r}=9\left\langle \epsilon \right\rangle$, the time $t_{\text{lucky}}\left(n=2\right)$ where the one-in-a-million fastest droplets surpass a given size n=2 decreases by about 50%. (*Bottom*) $t_{\text{lucky}}$ for different dissipation rates and as a function of the droplet size: we compare our DNS data to our framework with and without memory effects. The relative effects of memory on $t_{\text{lucky}}$ decrease with the volume-averaged dissipation rate and with increasing droplet size.

Our non-Markovian stochastic framework thereby enables a quantitative assessment of the effect of correlations on the growth of lucky droplets. To demonstrate this, we study the evolution of the probability of observing droplets with at least two collisions, i.e., n>2 (Fig. 3, *Top*). One can see that the data from the DNS (data points) is well approximated by our solution, Eq. **18**, with the empirical superposition of two Poisson processes, Eq. **9** (solid lines). When we turn off correlations (dashed lines), i.e., solving Eq. **18** only with the dominant Poisson process, we observe deviations at short times that become more pronounced with increasing volume-averaged dissipation. This implies that correlations can accelerate droplet growth on short timescales but the effect strongly depends on sufficiently high dissipation and number density (*SI Appendix*). However, the relative speedup of the $10^{-6}$ fastest growing droplets decreases significantly for larger droplet sizes, see Fig. 3(*Bottom*). Hence, we conclude that correlations can accelerate growth but can be considered small corrections for the growth of large droplets. This additionally solidifies the assumption that collisions are sufficiently rare to consider them as statistically independent events, see, e.g., refs. 17 and 19.

In the following, we thus focus on the effect of dissipation fluctuations alone, keeping in mind that the effect we find would be even larger when short-time correlations were included.

## Toy Model to Capture Dissipation Fluctuations

To estimate the effect of a time-dependent volume-averaged dissipation rate on the time it takes to bridge the size gap, we consider, as a simple toy model, an ensemble of small-scale cloud parcels with linear droplet growth. Conceptually, this is similar to the eddy-hopping approach, which models supersaturation fluctuations of cloud parcels just that here, we consider dissipation fluctuations instead of supersaturation fluctuations (43).

For each parcel, we model droplet growth by numerically solving a linear master equation, similar to Eq. **5**, but with a collision rate that depends on time to model the fluctuations in volume-averaged dissipation rates:

[21] $$\begin{array}{cc} {\dot{P}}_{n}\left(t\right) & =\lambda_{n-1}\left(t\right)P_{n-1}\left(t\right)-\lambda_{n}\left(t\right)P_{n}\left(t\right)\\ \lambda_{n}\left(t\right) & =g({\overset{¯}{\epsilon }}_{r}\left(t\right),n)\;. \end{array}$$

Here, we model the collision rates as a function g of droplet size and a slowly time-varying local dissipation rate (at fixed scale r), which is constrained in the following based on our DNS results.

**Fig. 4.**
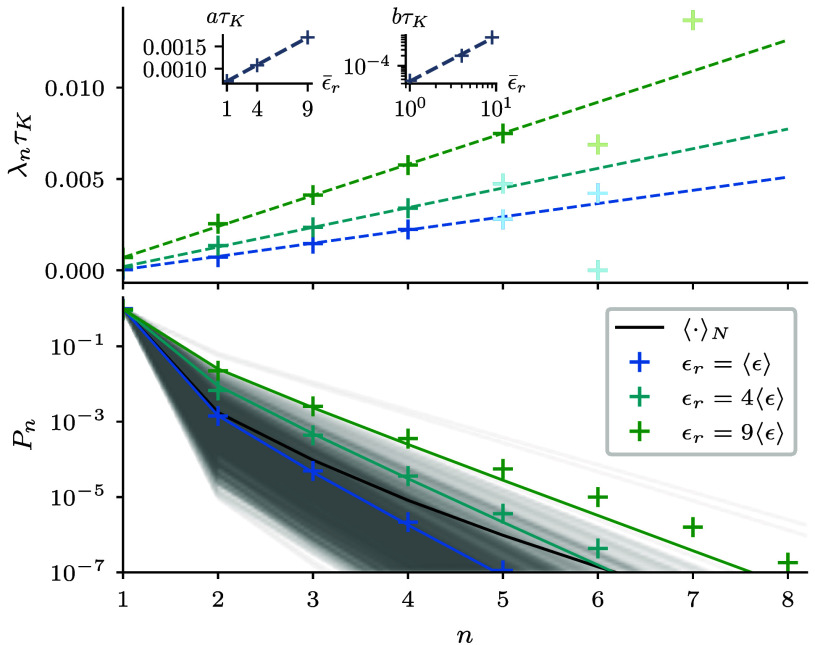
Time-dependent probability distribution of droplet sizes can be approximated by the toy model that integrates Eq. **5** with an effective $\lambda_{n}\left({\overset{¯}{\epsilon }}_{r}\right)$ parameterized by DNS (data points). (*Top*) Linear approximation of the collision rate as a function of collisions, where faded data points were excluded from the fit due to insufficient statistics. The *Inset* shows slope a and offset b of our linear approximation that are approximated by a linear function and power-law, respectively. (*Bottom*) Probability distribution at the end time of simulation to benchmark the toy model with numerical simulations. The toy model can be easily evaluated for an ensemble drawn from $P({\overset{¯}{\epsilon }}_{r})$ (gray lines) to find that the ensemble mean (black line) has a higher probability for larger droplets.

In general, the collision rates involve multiple processes (2) such as clustering (1, 44), sling effect (11, 45), and differential settling (9). We observe, however, an approximately linear dependence of the collision rate on the droplet size n, see (Fig. 4), albeit for very small n accessible to our numerical simulations. Hence, we make the crude and simplifying ansatz

[22] $$\begin{array}{c} g\left({\overset{¯}{\epsilon }}_{r},n\right)=a\left({\overset{¯}{\epsilon }}_{r}\right)\left(n-1\right)+b\left({\overset{¯}{\epsilon }}_{r}\right)\;, \end{array}$$

where the parameters depend on ${\overset{¯}{\epsilon }}_{r}$ (Fig. 4).

Based on our DNS results, we fit a to depend linearly on the volume-averaged dissipation $a=\left[1.23\left(2\right)\times 10^{-4}{\overset{¯}{\epsilon }}_{r}+6.0\left(2\right)\times 10^{-4}\right]\tau_{K}^{-1}$ and b as a power-law $b=3.5\times 10^{-5}\left(4\right){\overset{¯}{\epsilon }}_{r}^{1.33(8)}\tau_{K}^{-1}.$ The functional dependence of these parameters is, of course, rather a choice than a fit, given the few data points we have, but we believe it is sufficient that our toy model will capture the essence of how dissipation fluctuations may affect growth statistics. Indeed, Fig. 4(*Bottom*) shows that matching ${\overset{¯}{\epsilon }}_{r}$ with our reference simulations and numerically solving Eq. **5** (solid colored lines) yields good agreement with our numerical simulations (data points), where deviations in the tails are expected for larger ${\overset{¯}{\epsilon }}_{r}$ from neglecting correlations.

This toy model now allows us to rapidly generate droplet size distributions for a full ensemble of dissipation rates. Drawing dissipation rates from $P({\overset{¯}{\epsilon }}_{r})$ expected in clouds (cf. Fig. 1), we find that the ensemble mean has an increased probability for larger droplets compared to the mean dissipation (Fig. 4, *Bottom*). Since lucky droplets are statistical outliers, we can already conclude that the log-normal fluctuations in dissipation rate increase the size of lucky droplets. We are left with the question of whether this can significantly accelerate the growth of droplets of sufficient size to bridge the size gap.

Our toy model now provides an affordable approach to extend the evolution of droplet growth to longer times. On these timescales, however, the volume-averaged dissipation rate will fluctuate notably with an unknown timescale. A timescale can be estimated by dividing the energy contained in the flow by the dissipation rate. On the scale of an individual parcel, this yields a few seconds. Unfortunately, the precise timescales in clouds remain unknown because measurements of the volume-averaged dissipation rate on the relevant spatial scales (∼1 m) are currently not reachable within clouds (46). In the following, we assume a timescale of about $\tau_{\epsilon }=19\;\;\text{s}$ [we also tested 10 and 40 s with qualitatively similar results, see *SI Appendix* (39)], and construct time-dependent logarithmic dissipation rates as an Ornstein–Uhlenbeck process with corresponding variance and mean from the refined similarity hypothesis to recover the log-normal distribution $P({\overset{¯}{\epsilon }}_{r})$, Eq. **1**. We obtain this process by considering the logarithm of the dissipation ${\overset{¯}{\epsilon }}_{r}$, ξ (with ${\overset{¯}{\epsilon }}_{r}=e^{\xi (t)}$), as a Gaussian process with the following correlation property:

[23] $$\begin{array}{c} \langle \left(\xi \left(t\right)+\sigma^{2}/2\right)\left(\xi \left(t+\tau \right)+\sigma^{2}/2\right)\rangle =\sigma^{2}e^{-\tau /\tau_{\epsilon }}\;. \end{array}$$

**Fig. 5.**
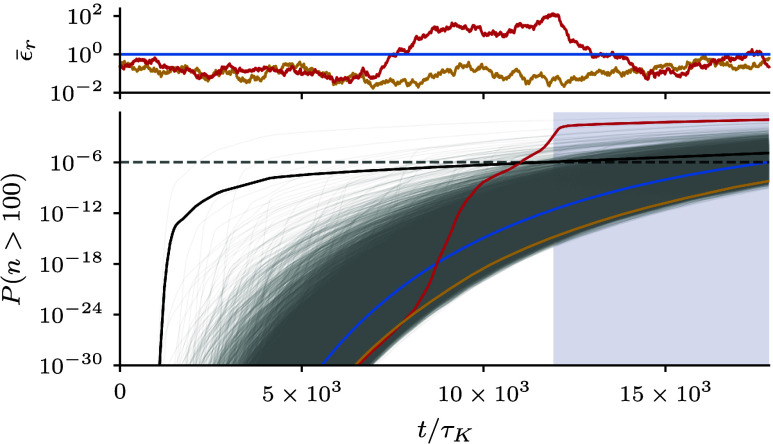
(*Top*) Two realizations of the normalized volume-averaged dissipation rate drawn from the same distribution—the one with the lowest and highest dissipation rate among $10^{5}$ randomly generated realizations. The corresponding dissipation rate may—as one of the example realizations shows—experience significant spikes. (*Bottom*) The probability that n>100, i.e., a droplet had at least 100 collisions as a function of time within our toy model, assuming a more realistic number density of $200\;\;{\text{cm}}^{-3}$. The $10^{5}$ ensemble realizations are shown in gray, whereas we have the ensemble mean in black. Notice that large outliers (logarithmic y-scale) dominate the mean, e.g., the high-dissipation realization (red) toward the end.

From the $10^{5}$ realizations that we generate, two are shown in Fig. 5 with minimal (yellow) and maximal (red) time-averaged dissipation ${\left\langle {\overset{¯}{\epsilon }}_{r}\right\rangle }_{t}\approx 1/5$ and ${\left\langle {\overset{¯}{\epsilon }}_{r}\right\rangle }_{t}\approx 7$, respectively, for a time series of about 40 times the timescale of the volume-averaged dissipation rate.

To estimate the effect of dissipation fluctuations on an ensemble of cloud parcels via Eq. **21**, we thus make our empirical ansatz Eq. **22** and model the time evolution of the volume-averaged dissipation ${\overset{¯}{\epsilon }}_{r}\left(t\right)$ as a stochastic process that is characterized by Eq. **23**. This way we obtain different realizations of parcels and their droplet growth (Fig. 5, *Bottom*). To consider a typical droplet number density of $200\;\;{\text{cm}}^{-3}$ instead of the upper bound $1,000\;\;{\text{cm}}^{-3}$ used in our main simulation, we rescale the collision rates proportionally to the density. Then, we can use our toy model to infer how much time is needed until lucky droplets bridge the size gap. Starting with an initial size corresponding to 12.5μm, it is on the order of 100 collisions that suffice to bridge the gap and reach about 50μm. Considering no fluctuations and only mean dissipation (blue), the fraction of droplets with more than 100 collisions would exceed the lucky-droplet threshold of $10^{-6}$ at about $18\times 10^{3}\tau_{K}$ (corresponding to 350 s, with the assumed cloud parameters).^†^ Considering the ensemble mean (black), representative of a full cloud, we would find the size gap to be bridged already at $12\times 10^{3}\tau_{K}$ (corresponding with the assumed cloud parameters to 230 s), implying that in our model with the chosen parameterization, dissipation fluctuations speed up rain formation by about 33% (see *SI Appendix* for controls). The individual realizations provide more insights into how this speed-up occurs: The ensemble average is dominated by a few statistical outliers. This is exemplified by looking at the ${\overset{¯}{\epsilon }}_{r}\left(t\right)$ realization with the highest mean dissipation rate (red), where one can see that, while initially even below mean conditions, the number of large droplets shoots up precisely when the dissipation rate features extreme peaks. This implies that rain droplet growth is drastically accelerated in cloud parcels with temporally high dissipation rates and that fluctuations of the volume-averaged dissipation could be crucial to bridge the size gap.

## Summary and Conclusions

In summary, we established a systematic approach to account for short-time correlations between individual collisions and spatiotemporal dissipation fluctuations. We showed that these short-time correlations can accelerate the growth of droplets on short timescales for high particle density and high dissipation, but that this is subleading to the overall acceleration due to fluctuations of the volume-averaged dissipation rate for large droplets. Using a toy model to capture dissipation fluctuations due to intermittency, we found that strong bursts of the volume-averaged dissipation rate can dominate the formation of fast-growing droplets to bridge the size gap much faster than assuming a constant volume-averaged dissipation rate. This underscores the potentially crucial contribution of dissipation fluctuations to bridge the size gap sufficiently fast for the onset of rain within 30 min in warm clouds.

## Supplementary Material

Appendix 01 (PDF)

## Data Availability

Files to reproduce the plots of the paper and code of the statistical model presented in the paper have been deposited in GitLab [https://gitlab.gwdg.de/tbaetge1/effects-of-correlated-collisions-and-intermittency-on-the-growth-of-lucky-droplets (47)]. The simulation data itself has been generated with our open-source Navier–Stokes solver TurTLE available at (https://gitlab.mpcdf.mpg.de/TurTLE/turtle) (48). The raw simulation data can be shared upon reasonable request.

## Footnotes

^*^Note that in our approach, we keep the full memory of all past collisions by means of a nested integration different from a single convolution with an effective memory kernel used in ref. 21.

^†^Note that this is less than 6 min, which is way below the onset time of rain of 30 min. Two reasons for such fast times are i) that we do not account for the time it takes to create droplets of 12.5 μm, and ii) that the collision efficiency for small droplets can be significantly reduced, thereby slowing down the collisional growth. While these factors could be, in principle, incorporated into our non-Markovian stochastic framework (similar to ref. 19 for the case of constant collision rates), they would require empirical information that still needs to be obtained. However, we expect that the qualitative results from our toy model will transfer to more realistic conditions.
