# Supplementary material for: Effects of correlated collisions and intermittency on the growth of lucky droplets
Source: Proc Natl Acad Sci U S A. 2026 Feb 23;123(9):e2502553123. doi: 10.1073/pnas.2502553123 (PMC12956867; doi:10.1073/pnas.2502553123)
Supplement: Supplementary file 1 — Appendix 01 (PDF) [file pnas.2502553123.sapp.pdf]

Supporting Information for

Effects of correlated collisions and intermittency on the growth of lucky droplets

Tobias Bätge, Johannes Zierenberg, and Michael Wilczek

E-mail: Michael.Wilczek@uni-bayreuth.de

This PDF file includes:

Supporting text
Figs. S1 to S5
Tables S1 to S5
SI References

Supporting Information Text

S1. Solution of evolution equation with constant collision rates

This section details how we solve the master equation, Eq. (5) in the main text. For completeness, we consider the master equation

$$\dot{P}_n^{\mathrm{M}}(t) = \lambda_{n-1} P_{n-1}^{\mathrm{M}}(t) - \lambda_n P_n^{\mathrm{M}}(t)\,, \qquad [1]$$

which describes the growth of droplets that collide with a constant rate in a monodisperse and statistically stationary background distribution. This can be solved by variation of constants: Here $\dot{P}_n^{M}(t) + \lambda_n P_n^{\mathrm{M}}(t) = 0$ is the homogeneous differential equation that is solved by $P_n^{M,\mathrm{hom}}(t) = ce^{-\lambda_n t}$. The method of the variation of constant with this homogeneous solution and the inhomogeneity $\lambda_{n-1} P_{n-1}^{\mathrm{M}}(t)$ then results in the following expression for the full solution

$$P_n^{\mathrm{M}}(t) = e^{-\lambda_n t}\left(c_n + \int_0^t \lambda_{n-1} P_{n-1}^{\mathrm{M}}(t_n) e^{\lambda_n t_n} dt_n\right) = c_n e^{-\lambda_n t} + \int_0^t \lambda_{n-1} e^{-\lambda_n (t-t_n)} P_{n-1}^{\mathrm{M}}(t_n)\, dt_n\,. \qquad [2]$$

The integration constant c_n is constrained by the initial condition $P_n^{\mathrm{M}}(0) = \delta_{n1}$ such that $c_1 = 1$ and $c_n = 0$ for $n > 1$.

Let us build up the solution according to this iterative pattern starting with $n = 1$. Here, the second term in Eq. (2) vanishes as there is no smaller size than $n = 1$. This yields, as one might expect, a simple exponential decay for $t \geq 0$. In preparation for our non-Markovian treatment, we can also write the Markovian solution with an integral extending over the full past and introduce a δ-influx to model the initial condition

$$P_1^{\mathrm{M}}(t) = e^{-\lambda_1 t} = \int_{-\infty}^t \delta(t_1) e^{-\lambda_1 (t-t_1)} dt_1\,. \qquad [3]$$

Following the above iterative pattern we obtain for $n = 2$ (recall $c_2 = 0$)

$$P_2^{\mathrm{M}}(t) = \int_{-\infty}^t \int_{-\infty}^{t_2} \delta(t_1) \lambda_1 e^{-\lambda_2 (t-t_2)} e^{-\lambda_1 (t_2 - t_1)} dt_1 dt_2\,, \qquad [4]$$

and $n = 3$

$$P_3^{\mathrm{M}}(t) = \int_{-\infty}^t \int_{-\infty}^{t_3} \int_{-\infty}^{t_2} \delta(t_1) \lambda_2 \lambda_1 e^{-\lambda_3 (t-t_3)} e^{-\lambda_2 (t_3 - t_2)} e^{-\lambda_1 (t_2 - t_1)} dt_1 dt_2 dt_3\,. \qquad [5]$$

Following this pattern further, the solution becomes

$$P_n^{\mathrm{M}}(t) = \int_{-\infty}^t \ldots \int_{-\infty}^{t_2} \delta(t_1) \left[\prod_{i=1}^{n-1} \lambda_i e^{-\lambda_i (t_{i+1} - t_i)}\right] e^{-\lambda_n (t - t_n)} dt_1 \ldots dt_n, \qquad [6]$$

which is provided in the main text.

S2. Parameters and simulation setup

In the foreseeable future, simulations cannot resolve the entire range of scales and complexity of cloud turbulence, where the integral scale is on the order of $\sim$ 100m while the Kolmogorov scale, depending on the dissipation rate, is on the order of 1mm.

Our approach to this challenge is to focus on smaller parcels of cloud turbulence that can be resolved within direct numerical simulations. Here, we use the pseudospectral fluid solver TurTLE (1) to create a statistically stationary flow of homogeneous isotropic turbulence in a periodic box. In that flow, we evolve the droplets according to Eq. (3) in the main text. Once the droplet distribution reaches a statistically stationary state, we allow droplets to merge upon collisions, where we assume a collision efficiency of one, see section S3. In total, our simulation box measures $\sim$ 860 Kolmogorov lengths in each direction (resolved on a 1024^3 grid). As the box covers a fixed number of Kolmogorov lengths, it may correspond to different physical dimensions depending on the assumed volume-averaged dissipation rate within the box. Due to intermittency, this volume-averaged dissipation rate fluctuates, which can be modeled by the refined similarity hypothesis, see Eq. (1) in the main text. As those fluctuations change the physical dimensions of the simulation box, we also have to vary the number of droplets per box accordingly to preserve the density, see Tab S2. Additionally, the fluctuations also influence the non-dimensional characteristics that govern the role of inertia and gravity on the dynamics of the droplets, Stokes number and Froude number, respectively, as sketched in Fig. 1 in the main text.

For our simulations, we choose a mean dissipation rate and a number concentration of droplets at the upper end of the parameter range that is typically observable within clouds (2), see Tab. S1. We initialize the droplets as a monodisperse population, corresponding to a size of $12.5\,\mu$m, which is in the range of typical droplet sizes. To capture typical fluctuations of the dissipation rate on the scales of the simulation box, we once take the volume-averaged dissipation rate of the simulation corresponding to the global mean as well as four and nine times higher, see Tab. S2. As discussed above, the effective volume of our box changes accordingly. So while for $\epsilon_r = 9\langle\epsilon\rangle \approx 0.36\mathrm{m}^2\mathrm{s}^{-3}$ the simulation box has a length corresponding approximately to a quarter meter, for $\epsilon_r = \langle\epsilon\rangle \approx 0.04\mathrm{m}^2\mathrm{s}^{-3}$ the box length corresponds to almost half a meter. According to the effective volume, the number of droplets in the simulation box N_{sim} also varies.

mean dissipation rate $\langle\epsilon\rangle$ [m^{2}s^{-3}]	viscosity ν [m^{2}s^{-1}]	droplet radius a [μm]	number density ρ_{N} [N/cm^3]	density ratio water/air ρ_L/ρ_0
$0.001 - 0.04$	$\approx 1.5 \times 10^{-5}$	< 20	$100 - 1000$	≈ 800

Table S1. Typical literature values which govern droplet and turbulence properties (2): mean dissipation ϵ, viscosity ν, droplet radius a, number density n and density ratio between water and air ρ_L/ρ_0

#	ϵ_r $\left[\frac{\mathrm{m}^2}{\mathrm{s}^3}\right]$	a [μm]	ρ_{N} $\left[\frac{N}{\mathrm{cm}^3}\right]$	St$_r$	Fr$_r$	ρ_{N} $\left[\frac{N}{\eta^3_{K,r}}\right]$	N_{sim}
1	0.04	12.5	1000	0.1	0.15	1.8×10^{-1}	115×10^6
2	0.16	12.5	1000	0.2	0.41	6.3×10^{-2}	40×10^6
3	0.36	12.5	1000	0.3	0.76	3.5×10^{-2}	22×10^6

Table S2. Assumed physical parameters such as volume-averaged dissipation rate, droplet size, and number density in terms of the microscale and the total number of droplets in the simulation domain, and corresponding non-dimensional characteristics. The characteristics of the flow remain unchanged in code units; all runs are conducted on a 1024^3 grid with a spatial resolution of $k_{\mathrm{max}}\eta \approx 3$ and a Reynolds number of Re$_\lambda \approx 200$.

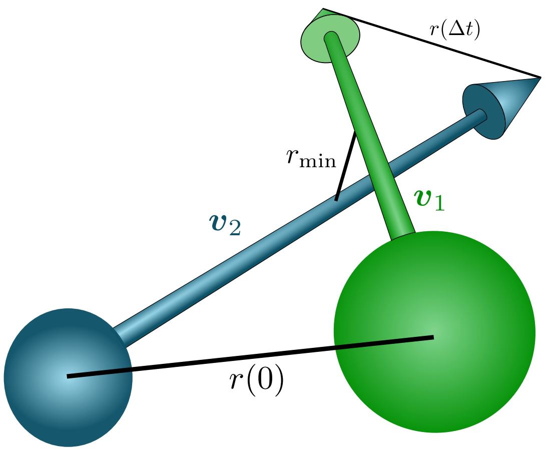

Fig. S1. Two particles with velocities $\boldsymbol{v}_{1/2}$ and separation $r(0)$ at the beginning of the timestep. Their separation changes then – according to the linear extrapolation – to $r(\Delta t)$ within one timestep, whereas it may reach the minimal distance, r_{min} in between.

S3. Collision procedure

For the detection of collisions of larger particles, it could be simply sufficient to check whether particles overlap at the corresponding timesteps. For an example that uses this approach in our code TurTLE, we refer to (3). In that case, the procedure is justified as particles may not move relative to each other more than the collision radius within one timestep. However, the droplets we consider are small, and through the described procedure one might miss collisions in between timesteps. Therefore, we linearly extrapolate the trajectories every timestep to check for particles coming closer than the collision radius between timesteps (as also done, e.g., in (4)). We describe the procedure in the following: Numerically detecting collisions requires first identifying droplets that may collide within one timestep. The fluid solver TurTLE (1) features functionality that efficiently provides all pairs of particles below a certain distance, in the following referred to as cutoff distance. Here, we take the grid spacing as the cutoff distance for our particles, which are significantly smaller than that. Given a sufficiently small timestep, they do not travel distances larger than the grid spacing within a single timestep. Having identified the collision candidates, we can extrapolate their trajectories to check for collisions between the current and next timestep. Here, linear extrapolation is sufficient as the small numerical errors do not accumulate (This is in contrast to the time evolution itself, where small errors grow exponentially).

Let two collision candidates have the radii $r_{p,1}$ and $r_{p,2}$, positions $\boldsymbol{x}_1$ and $\boldsymbol{x}_2$, and velocities $\boldsymbol{v}_1$ and $\boldsymbol{v}_2$ at the time $t = 0$. Via extrapolation, we can check for collisions occurring during the following timestep Δt. For this, we need to find the minimal distance between the particle centers by considering

$$r_{\text{min}}^2 = \min_{\Delta t \geq t > 0} \left[r(t)^2 \right] = \min_{\Delta t \geq t > 0} \left[\left| (\boldsymbol{x}_1 + \boldsymbol{v}_1 t) - (\boldsymbol{x}_2 + \boldsymbol{v}_2 t) \right|^2 \right] . \qquad [7]$$

Hence, the following condition determines the time when the droplets reach their minimum distance:

$$\frac{d}{dt} \left[(\boldsymbol{x}_1 + \boldsymbol{v}_1 t) - (\boldsymbol{x}_2 + \boldsymbol{v}_2 t) \right]^2 = 0 \quad \Rightarrow \quad \boldsymbol{r} \cdot \Delta \boldsymbol{v} + (\Delta \boldsymbol{v})^2 t = 0 \qquad [8]$$

which implies

$$t_{\text{min}} = -\frac{\boldsymbol{r} \cdot \Delta \boldsymbol{v}}{(\Delta \boldsymbol{v})^2} . \qquad [9]$$

Here, $\boldsymbol{r} = \boldsymbol{x}_1 - \boldsymbol{x}_2$ is the relative distance of the particles and $\Delta \boldsymbol{v} = \boldsymbol{v}_1 - \boldsymbol{v}_2$ their relative velocity. Depending on the time t_{min}, when the droplets reach the minimal distance, we can exclude or conclude that a collision will happen within the next timestep. For $\Delta t \geq t_{\text{min}} > 0$ we have a collision, if $r_{\text{min}} = r(t_{\text{min}})$ fulfills the collision condition,

$$r_{\text{min}} < r_{p,1} + r_{p,2} . \qquad [10]$$

Using the expression for t_{min}, Eq. (9), we obtain:

$$r_{\text{min}} = |\boldsymbol{r}(0) + \Delta \boldsymbol{v} t_{\text{min}}| = \sqrt{\boldsymbol{r}^2 - \frac{(\boldsymbol{r} \cdot \Delta \boldsymbol{v})^2}{(\Delta \boldsymbol{v})^2}} . \qquad [11]$$

For $t_{\min} > \Delta t$, we can also have a collision. While the particles reach their minimal distance only at a later point, their distance may become smaller than the collision radius during the timestep. To confirm this case, we check if at the end of the timestep, the distance $r(\Delta t)$ fulfills the collision condition:

$$r(\Delta t) < r_{p,1} + r_{p,2}\,. \qquad [12]$$

If one of the two cases above is fulfilled we conclude that during the timestep a collision occurs. Once we detect a collision, we model the process of the collision itself. Here, we change the mass and velocity of the colliding particles according to momentum and mass conservation upon a collision, see Fig. S2, where we choose the droplet with the smaller index to grow: The position changes to the center of mass of the two colliding particles. This can be summarized as:

$$m_j \to m_i + m_j \quad \boldsymbol{v}_j \to \frac{m_i \boldsymbol{v}_i + m_j \boldsymbol{v}_j}{m_i + m_j} \quad \boldsymbol{x}_j \to \frac{m_i \boldsymbol{x}_i + m_j \boldsymbol{x}_j}{m_i + m_j} \quad \text{with} \quad j < i\,. \qquad [13]$$

We add the droplet with the larger index to a list of particles, which we delete before the next timestep. A consistent choice of which particle we delete simplifies the bookkeeping, while it does not make a physical difference.

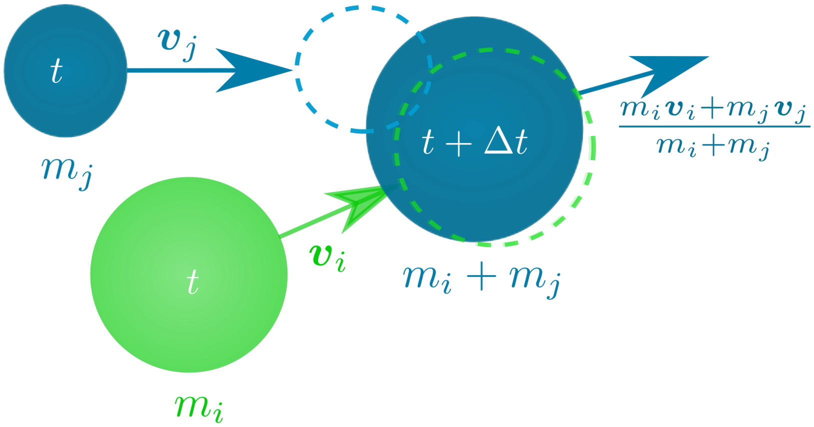

Fig. S2. Upon collision of particle j and i (with $i > j$) within one timestep Δt, they merge while mass and momentum are conserved. According to the collision procedure, the droplet with the smaller index persists while we delete the other one.

S4. Survival functions for larger droplet sizes

In Fig. 2 of the main text, we show the extracted survival functions and a fit based on the superposition of two Poisson processes. Consequently, we approximate the different survival probabilities as a sum of two exponentials:

$$S_n(\tau) = A_n^{\mathrm{slow}} e^{-\lambda_n^{\mathrm{slow}}\tau} + A_n^{\mathrm{fast}} e^{-\lambda_n^{\mathrm{fast}}\tau} . \qquad [14]$$

Here, $\lambda_n^{\mathrm{slow}}$ denotes the collision rate capturing the exponential tail of the survival probability and the long-time behavior while $\lambda_n^{\mathrm{fast}}$ corresponds to the rate of the additional short-time process. A_n^{slow} and A_n^{fast} quantify the respective weights of the two processes. The fit values up to n=4 are shown in Tab. S3, and form the basis of the solutions P_3 and P_4 in Fig. 3 of the main text according to Eq. (19) in the main text.

	$\epsilon_r = \langle\epsilon\rangle$	$\epsilon_r = 4\langle\epsilon\rangle$	$\epsilon_r = 9\langle\epsilon\rangle$
$\lambda_1^{\mathsf{slow}}$ $[\tau_K^{-1}]$	$3.616(8) \times 10^{-5}$	$1.999(4) \times 10^{-4}$	$7.00(1) \times 10^{-4}$
A_2^{fast}	0.298(2)%	0.718(2)%	1.322(3)%
$\lambda_2^{\mathsf{slow}}$ $[\tau_K^{-1}]$	$7.211(2) \times 10^{-4}$	$1.3552(6) \times 10^{-3}$	$2.548(2) \times 10^{-3}$
$\lambda_2^{\mathsf{fast}}$ $[\tau_K^{-1}]$	$1.4(2) \times 10^{-1}$	5.39(5)	8.05(9)
A_3^{fast}	0.298(4)%	0.78(1)%	1.294(3)%
$\lambda_3^{\mathsf{slow}}$ $[\tau_K^{-1}]$	$1.459(5) \times 10^{-3}$	$2.356(3) \times 10^{-3}$	$4.117(4) \times 10^{-3}$
$\lambda_3^{\mathsf{fast}}$ $[\tau_K^{-1}]$	2.1(1)	2.17(8)	5.02(7)
A_4^{fast}	0.000(4)%	0.36(1)%	0.527(6)%
$\lambda_4^{\mathsf{slow}}$ $[\tau_K^{-1}]$	$2.238(3) \times 10^{-3}$	$3.401(4) \times 10^{-3}$	$5.761(3) \times 10^{-3}$
$\lambda_4^{\mathsf{fast}}$ $[\tau_K^{-1}]$	-	2.1(5)	3.7(3)

Table S3. Fit parameters for the survival functions in Fig. S3 for the three different parameter sets. $\lambda_1^{\mathsf{slow}}$ corresponds to the exponential decay rate of the first size. Here we only have one exponential as the first collision cannot correlate with a previous one. Otherwise, we fitted a superposition of a short and long-time process. $\lambda_i^{\mathsf{slow}}$ corresponds to the long-time collision rate while $\lambda_i^{\mathsf{fast}}$ corresponds to the rate of the additional short-time process. Here, $A_i^{\mathsf{fast}} = 1 - A_i^{\mathsf{slow}}$ denotes the weight of the short-time process and quantifies the fraction of droplets colliding due to correlations.

In the main text (Fig. 4), we also compare the solution for the droplet population without memory effects, i.e., the solution to the Markovian master equation, $\dot{P}_n(t) = \lambda_{n-1}P_{n-1}(t) - \lambda_n P_n(t)$, constrained by collision rates from the DNS data for a range of droplet sizes. Accordingly, we need the constant collision rates neglecting the effect of correlations, i.e., the rate corresponding to the exponential tail of the survival function, also for higher values of n. For higher values of n, we only have a few droplets reaching those sizes and an insufficient statistical basis to fit those values. Figure S3 also includes the less converged survival probabilities for higher n complementary to Fig. 2 in the main text. In Fig. 4 in the main text, we consider the fit for $\lambda_n^{\mathrm{slow}}$ up to a value of n where individual collisions lead to fluctuations featured as pronounced steps and the fit becomes unreliable. For higher values of n, we therefore extrapolate.

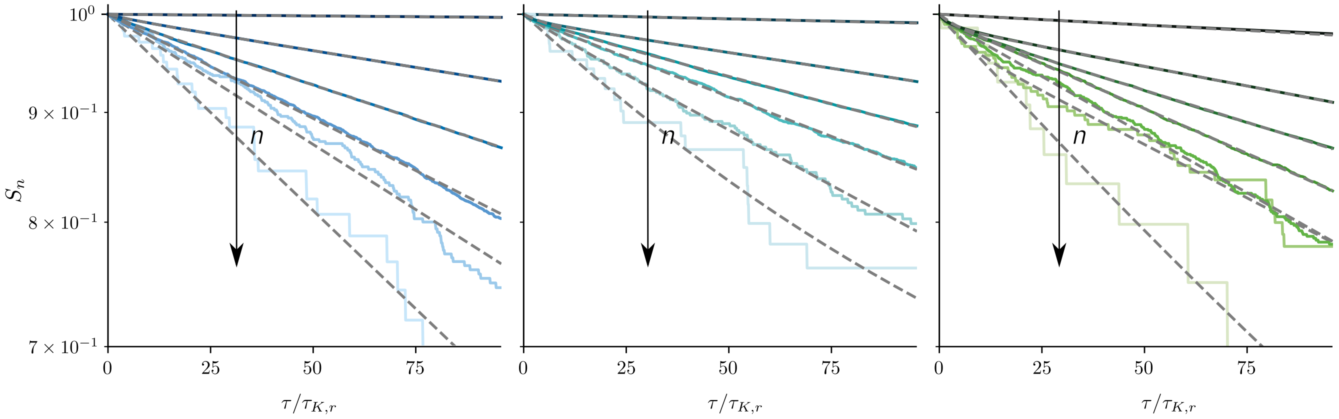

Fig. S3. Survival probabilities for the different normalized volume-averaged energy dissipation rates $\bar{\epsilon}_r = 1, 4, 9$ from left to right. With increasing droplet size n, the lines become lighter and statistically less converged. In Fig. 4 of the main text, we extrapolate the values of the collision rates $\lambda_n^{\mathsf{slow}}$ associated with the exponential tail.

S5. Memory effects in DNS with lower droplet number density

Let us consider the memory effects in collisional droplet growth for a lower droplet number density of $400\,\mathrm{cm}^{-3}$. We start with the collision rates and survival functions as shown in Fig. S4. Generally, with a lower number of droplets, collisions are significantly less likely and, therefore, statistical convergence is weaker as one can also see by the larger scatter compared to Fig. 2 in the main text. As a consequence, we only show the collision rates and survival functions of the first three sizes. The collision rates still have a clear signature of correlations between consecutive collisions, more pronounced in the case of $\epsilon_r = 9\langle\epsilon\rangle$. As for the case of higher number density, the survival functions in the case $\epsilon_r = \langle\epsilon\rangle$ are almost exponential, i.e., memory effects are negligible. In contrast, the survival probabilities for $\epsilon_r = 9\langle\epsilon\rangle$ deviate at short times from an exponential behavior through a more rapid decrease. The fit of the superposition of two exponential functions – one capturing the tail and one the memory effects at short times – shows that the weight of the short-time process $A_i^{\mathrm{fast}} \sim 0.52 - 0.57\%$ is still notable. However, its relevance compared to the case studied in the main text is further reduced. For the fit parameters, we refer to Tab. S4.

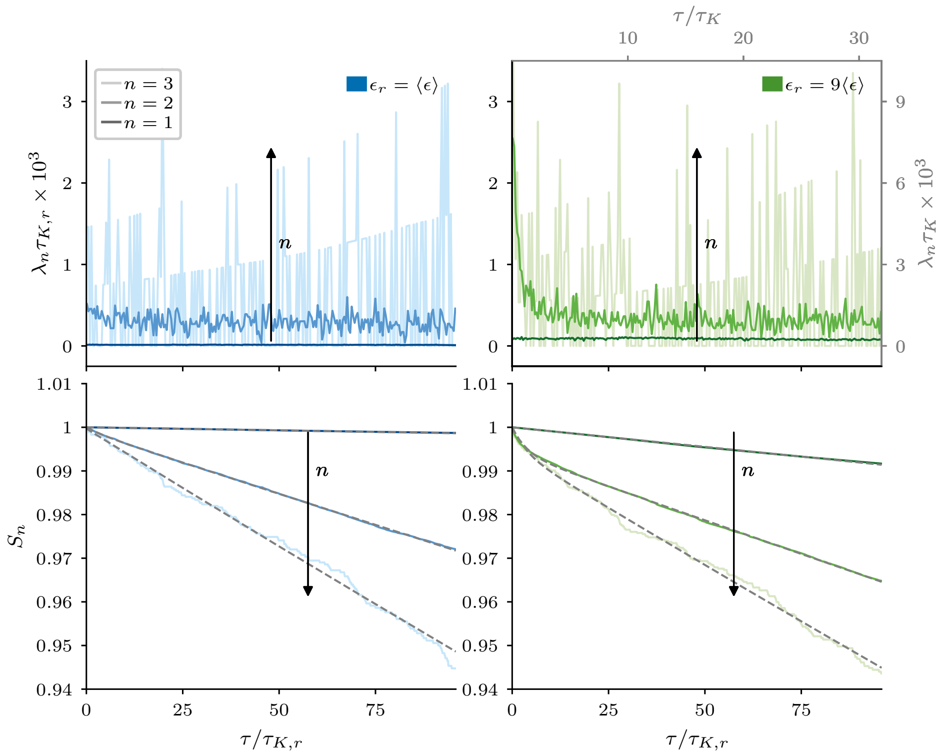

Fig. S4. Collision rates λ_n and survival probability S_n for different sizes n for a number density of $400\,\mathrm{cm}^{-3}$: here, τ denotes the time since the last collision except for $n = 1$, where it is the time since the simulation started. (Top) Except for λ_1, which is close to constant, all collision rates are, for short times, significantly increased before they reach approximately a constant (up to fluctuations). The relative increase on short times is more pronounced for $\epsilon_r = 9\langle\epsilon\rangle$ than for $\epsilon_r = \langle\epsilon\rangle$, while generally, collision rates are higher for a higher volume-averaged energy dissipation rate. (Bottom) We fit each survival function by superposing two exponentials, one for the tail and one to capture short-time correlations. For the fit parameters, see Tab. S4. (Bottom left) For the mean volume-averaged dissipation rate $\epsilon_r = \langle\epsilon\rangle$, the survival probabilities all have a shape close to an exponential one. The fast Poisson process has only a weight between 0-0.569%. (Bottom right) For $\epsilon_r = 9\langle\epsilon\rangle$, one can visually see the effect of correlations for short times where the fast Poisson process weights up to 0.569%.

#	$\epsilon_r = \langle\epsilon\rangle$	$\epsilon_r = 4\langle\epsilon\rangle$	$\epsilon_r = 9\langle\epsilon\rangle$
$\lambda_1^{\mathrm{slow}}\,[\tau_K^{-1}]$	$1.43(5) \times 10^{-5}$	$8.02(2) \times 10^{-5}$	$2.699(2) \times 10^{-4}$
A_2^{fast}	$0.086(2)\%$	$0.285(1)\%$	$0.52(1)\%$
$\lambda_2^{\mathrm{slow}}\,[\tau_K^{-1}]$	$2.903(3) \times 10^{-4}$	$5.468(5) \times 10^{-4}$	$9.498(9) \times 10^{-4}$
$\lambda_2^{\mathrm{fast}}\,[\tau_K^{-1}]$	$2.2(2) \times 10^{-1}$	$4.15(8) \times 10^{-1}$	$8.2(2) \times 10^{-1}$
A_3^{fast}	$0.031(9)\%$	$0.000(7)\%$	$0.569(2)\%$
$\lambda_3^{\mathrm{slow}}\,[\tau_K^{-1}]$	$5.47(2) \times 10^{-4}$	$1.09(3) \times 10^{-3}$	$1.61(5) \times 10^{-3}$
$\lambda_3^{\mathrm{fast}}\,[\tau_K^{-1}]$	$4(9)$	-	$8.0(4) \times 10^{-1}$

Table S4. For $\epsilon_r = \langle\epsilon\rangle$, $\epsilon_r = 4\langle\epsilon\rangle$ and $\epsilon_r = 9\langle\epsilon\rangle$ and a number density of $400\mathrm{cm}^{-3}$, the table displays fit parameters for the survival functions in Fig. S4. $\lambda_1^{\mathrm{slow}}$ corresponds to the exponential decay rate of the first size. Here we only have one exponential as the first collision cannot correlate with a previous one. Otherwise, we fitted a superposition of a short and long-time process. $\lambda_i^{\mathrm{slow}}$ corresponds to the long-time collision rate while $\lambda_i^{\mathrm{fast}}$ corresponds to the rate of the additional short-time process. Here, $A_i^{\mathrm{fast}} = 1 - A_i^{\mathrm{slow}}$ denotes the weight of the short-time process and quantifies the fraction of droplets colliding due to correlations.

As one might expect, the collision rates $\lambda_n^{\mathrm{slow}}$ decrease in good approximation linearly with the density. This can be verified

by comparing the ratio of the estimated collision rates of our two simulated conditions, density 400cm^{-3} (here, Tab. S4) and density 1000cm^{-3} (main text, Tab. S3), which approximately yields the density ratio 0.4, see Tab. S5. We use this proportionality in the construction of the toy model to consider also number densities of 200cm^{-3}.

	$\epsilon_r = \langle\epsilon\rangle$	$\epsilon_r = 4\langle\epsilon\rangle$	$\epsilon_r = 9\langle\epsilon\rangle$
$\lambda_1^{\text{slow,A}}/\lambda_1^{\text{slow,B}}$	0.40	0.40	0.39
$\lambda_2^{\text{slow,A}}/\lambda_2^{\text{slow,B}}$	0.40	0.40	0.37
$\lambda_3^{\text{slow,A}}/\lambda_3^{\text{slow,B}}$	0.38	0.46	0.39

Table S5. Ratios of λ_n^{slow} from Table S4 (A) to Table S3 (B) for $n = 1, 2, 3$ and different ϵ_r.

S6. Sensitivity analysis of the toy model

Here we change the correlation time τ_ϵ of the random process within the toy model, which models the fluctuations of the volume-averaged dissipation in time. By taking approximately half and double the correlation time, we test how sensitive our results are in that regard. In the main text, we reported a speed-up to bridge the size gap, i.e of the one in a million fastest growing droplets to collide at least 100 times, of 33% relative to mean conditions with a correlation time τ_ϵ corresponding to about 19s with the assumed physical parameters, see Sec. S2. With about half the correlation time, the speedup reduces to 17%, and with roughly double the correlation time, it increases to 56%, see Fig. S5. This shows that the precise value of the speed-up is rather sensitive to the correlation time of the local dissipation fluctuations. However, the qualitative result of a speed-up is also present for other plausible correlation times.

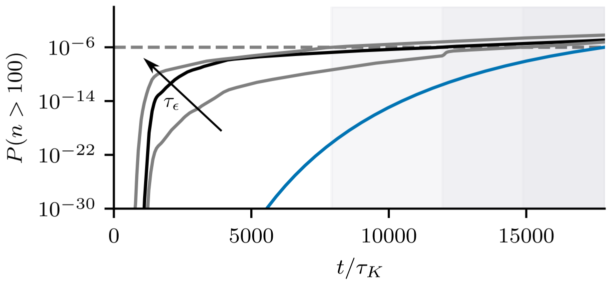

Fig. S5. Probability of a droplet with more than a hundred collisions as a function of time. The blue line corresponds to mean conditions, and the black line corresponds to the ensemble average over 10^5 realizations of the toy model as presented in Fig. 5 of the main text. The two dark gray lines correspond to the ensemble average over 10^5 realizations of the toy model with approximately twice and half the correlation time for the local dissipation fluctuations compared to the one in the main text. The gray shaded areas mark the crossings of the 10^{-6} threshold.

References

1. CC Lalescu, B Bramas, M Rampp, M Wilczek, An efficient particle tracking algorithm for large-scale parallel pseudo-spectral simulations of turbulence. *Comput. Phys. Commun.* **278**, 108406 (2022).
2. H Pruppacher, J Klett, *Microphysics of Clouds and Precipitation*, Atmospheric and Oceanographic Sciences Library. (Springer Netherlands, Dordrecht) Vol. 18, (2010).
3. JA Arguedas-Leiva, J Słomka, CC Lalescu, R Stocker, M Wilczek, Elongation enhances encounter rates between phytoplankton in turbulence. *Proc. Natl Acad. Sci. USA* **119**, e2203191119 (2022).
4. T Bhowmick, M Iovieno, Direct numerical simulation of a warm cloud top model interface: Impact of the transient mixing on different droplet population. *Fluids* **4**, 144 (2019).
